# Preuse Acceptance of a Family-Centered, Need-Based, and Interprofessional Perinatal Care Mobile Health Intervention: Exploratory Study

**DOI:** 10.2196/66658

**Published:** 2025-06-12

**Authors:** Kristina Killinger, Verena Seyfried, Katharina Brusniak, Markus Wallwiener, Michael Abou-Dakn, Dorothea Scholle, Stephanie Wallwiener

**Affiliations:** 1 Department of Gynecology and Obstetrics, University Hospital Heidelberg, Im Neuenheimer Feld 440, Heidelberg, 69120, Germany, 49 62215632078; 2 Department of Anesthesiology and Intensive Care Unit, Florence-Nightingale-Hospital, Duesseldorf, Germany; 3 Department of Gynecology, University Hospital Halle, Halle, Germany; 4 Department of Obstetrics and Gynecology, St. Joseph's Hospital Berlin, Berlin, Germany; 5 Department of Obstetrics and Perinatal Medicine, University Hospital Halle, Halle, Germany

**Keywords:** perinatal, mobile app, need-based care, patient empowerment, preuse acceptance, family, digital health, smartphone, eHealth, preterm birth, woman, Germany, psychosocial support, parenting, survey, telehealth, telemedicine

## Abstract

**Background:**

The perinatal period is one of the most vulnerable times a woman experiences. Multidimensional, interprofessional, and personalized support is needed to improve outcomes in women’s and children’s health while strengthening partner relationships at the same time. Although a vast amount of support services already exist in Germany for psychosocial counseling during the perinatal period, groups who are especially at risk do not take advantage of them.

**Objective:**

Family eNav is an app-based intervention developed by experts in the field of medical and psychosocial support to help young parents navigate through primary and secondary care services in Germany according to their needs. It also empowers patient and parenting perspectives through self-education and symptom monitoring for different settings, for example, mental health and preterm birth. While the intervention will be evaluated in a multicenter, randomized, controlled trial, the focus here lies on the conception of the app, demand among patients, and preuse acceptance.

**Methods:**

During the conception phase, we conducted an explorative study with prospective users and experts in the perinatal psychosocial field to understand the need and preuse acceptance of the intervention. We interviewed 20 participants with a semistructured guide, analyzing their responses using systematic text condensation. Additionally, we conducted a short survey on general questions concerning digitalization within the health care system among the participants.

**Results:**

We established two main themes: (1) access and barriers to health care and psychosocial services and (2) high preuse acceptance of app-based intervention. Health care and psychosocial providers indicated that there is a high demand for their services, which cannot always be met immediately, and at the same time, they are doubtful of reaching those individuals most in need. Prospective users and health and social care providers alike showed great interest in the perinatal navigator and suggested a variety of needs and content requirements to be included. Regionality, availability, and individualized content were underlined as success factors for high user acceptance. Barriers consisted of data protection concerns, as well as denial of their own needs.

**Conclusions:**

Our findings show great acceptance for an app-based intervention on the part of both prospective users and service providers. Feedback on requirements and content, as well as possible barriers, was taken into consideration while developing the app.

## Introduction

Pregnancy and early parenthood represent a turning point in life. Sufficient multidimensional support during pregnancy may be beneficial for mother-child relationships [1,2], whereas a lack of support can lead to manifold psychosocial risk factors [3]. Exposure to psychosocial risk factors, such as maternal depression, stress, and low social support, negatively affects pregnancy and child development [4,5]. Maternal and children’s health and social inequality are tightly linked [6-9]. Therefore, improving maternal psychosocial risk factors during this phase represents an important public health aim.

In 2015, a cross-sectional survey in Germany of over 7500 families with small children showed that around 40% of families have three and more stressors, consisting of biographic, perinatal, and psychosocial measures, cumulatively, whereby at least 25% of participants reported that the parenting role was associated with stress [10]. A vast amount of support services already exist in Germany. The ministry for families, seniors, women, and children even offers web-based services through a family portal [11]. For families in troublesome situations, early preventative measures are outlined, from diminishing exposure to violence to promoting a positive environment for development, which are all compiled—and subsidized—within the initiative called “Frühe Hilfen” (translation: Early Help) [12]. Frühe Hilfen conducted several studies on how socioeconomic factors are related to obstacles to using early childhood prevention services. They found that families from lower socioeconomic backgrounds knew less about assistance programs in general and were less likely to actively make use of them. Programs such as home visits or family midwives, who provide support during the first year after childbirth, were more often used by families with lower socioeconomic backgrounds because they were offered to them on the basis of need, and parents did not have to actively look for them [13,14].

Women show a high tendency to search for pregnancy-related information on the web and via smartphone apps [15,16]. The number of health apps for pregnant women that are easy to access and can introduce new ways to perinatal care delivery, especially in low socioeconomic settings, is growing [15,17]. The quality and effectiveness of mobile apps vary tremendously, though, which can lead to mistrust and discontinuance of the app [18,19]. Nonetheless, multiple randomized controlled trials (RCTs) showed health-promoting effects of using mobile health (mHealth) apps, for example, in weight management, gestational diabetes management, and maternal mental health [20,21]. Introducing a digital tool that has been developed with a user- and provider-centered approach opens a realistic opportunity to find evidence-based information and also lowers the threshold for gaining access to this information by digitally interlinking families in need with their regional service providers.

Our research project, Family eNav, is a multicenter health service program funded through the innovation funds of the Joint National Committee (German: Gemeinsamer Bundesausschuss). It was our objective to develop an app-based perinatal guide to better coordinate need-based primary and secondary prevention care and empower patient and parenting perspectives through self-education and symptom monitoring. Intensifying the link between health and social system support through an app-based navigator may ameliorate care for pregnant women and young families, especially those with psychosocial risk factors, and hence may lead to higher quality of life, relieve stress, and strengthen partner and parent-child relationships.

In the conception phase, we tried to develop the content basis for the guide by evaluating the demand for and acceptance of such an app-based intervention. The aim of this study was therefore to gain a deeper understanding of young families’ needs and reasons for using services to establish the demand for an app-based perinatal guide. At the same time, we tried to shed light on the provider’s perspective on how to successfully integrate an app-based perinatal navigator to determine factors of success and possible barriers.

## Methods

### Study Design

The study was conducted at the University Hospital of Heidelberg with pregnant women and young mothers, as well as health care experts [22]. Study participants were recruited between November 2021 and March 2022. We used purposive sampling to include all perspectives necessary for the conception of an app-based perinatal guidance program. Overall, we recruited 9 prospective users, among whom 5 users were pregnant and 4 users had infants, and 13 experts from all areas of perinatal services (Table 1). Eligibility criteria for users were older than 18 years of age and fluency in German. To be eligible for participation, service providers had to be involved in the care of pregnant women or young families. We conducted interviews until information saturation was reached.

**Table 1.** Study participant information.

	Age (years)	Highest level of education	Duration of interview (min)
Patient			
Pregnant in week 32 (interview 1)	34	University degree	18
Pregnant in week 28 (interview 2)	32	University degree	33
Pregnant in week 20 (interview 3)	30	Secondary level	21
Mother of 6-week-old infant (interview 4)	29	University degree	19
Mother of 4-month-old infant (interview 5)	31	Secondary level	29
Mother of 8-week-old infant (interview 6)	29	Secondary level	63
Mother of 3-month-old twins (interview 7)	30	University degree	38
Pregnant in week 11 (interview 8)	33	Secondary level	29
Pregnant in week 12 (interview 9)	31	Vocational training	17
Expert			
Course teacher (interview 10)	34	University degree	21
Psychologist or psychotherapist (interview 11)	65	University degree	30
Physiotherapist (interview 12)	27	Vocational training	29
Midwife (interview 13)	61	Vocational training	28
Coordinator at Frühe Hilfen (interview 14)	48	University degree	38
Pediatrician (interview 15)	62	Medical examination	36
Professor of medical psychology (interview 16)	46	Habilitation	24
Preterm development aid worker (interview 17)	41	University degree	45
Frühe Hilfen (interview 18)	56	University degree	43
Frühe Hilfen (interview 19)	37	University degree	29
Head of Gynecology Department (interview 20)	64	Medical degree	59

Semistructured interviews with a mean length of 32.45 (SD 9.54) minutes were held by 3 trained investigators following a previously established interview guide with primarily open-ended questions. At the end of the semistructured interviews, we added a general survey of the participants’ perspective on digital health with primarily closed-ended questions, using a scaling system (Multimedia Appendix 1). We performed systematic text condensation on the responses to the questions, which is a descriptive method concentrating on people’s experiences rather than the underlying meaning [23]. Through meaning units, we established a coding scheme to form predominant themes in accordance with our research questions. Researcher triangulation was used to increase reliability. Specific quotes were translated into English to underline the main perspectives. The answers to the digital health survey were assessed through descriptive statistics.

The study aimed to explore the perceived status quo of perinatal services and investigate whether there was a demand for a digital navigator, and also determine preuse acceptance of the planned mHealth intervention study [24]. Second, we wanted to give our prospective users and collaborators a voice within the conception phase of the app. Data were analyzed descriptively.

### Ethical Considerations

The study was approved by the ethical committee of the University of Heidelberg (S-344/2022). Informed consent was gathered prior to the interviews. Participants gave oral and written permission to record the interviews and perform data analyzation. They were later transcribed verbatim. The data were anonymized. Participants did not receive any compensation.

## Results

### Overview

**Figure 1.**
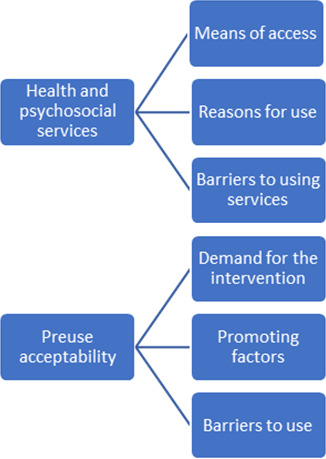
Main themes and categories retrieved from 20 interviews during the conception period of an app-based intervention study, called Family eNav.

Through our analysis, we established the following main themes: health care and psychosocial services, and preuse acceptance of an app-based intervention (Figure 1). We listed the most important meaning units for preuse acceptance by comparing the views of our prospective users and experts.

### Health Care and Psychosocial Services: Access and Barriers

#### Means of Access to Psychosocial and Health Care Services

Internet-based methods, such as search engines, social media, and local home pages to access perinatal services, made up at least 50% of the responses from users and experts. Most experts said they were contacted via email. Conventional methods, such as the telephone or the formal referral system, made up the rest. Numerous experts additionally stressed the importance of word-of-mouth recommendations.

*I mainly google things or ask colleagues who already were pregnant for recommendations*.

[Interview 2]

#### Reasons for Using and Not Using Services

The willingness to take advantage of health care services during and after the pregnancy was high in our study group. Almost all participants at least tried to take part in activities such as antenatal classes, yoga for pregnant women, baby swimming classes, or courses for family bonding. Reasons for not taking advantage of services were a lack of interest and time, or low perceived value of the service itself, or low perceived qualification of the personnel, lack of knowledge, and higher barriers due to COVID-19 restrictions on services. Some minimized the use of certain services because of disadvantages due to cost and organizational load. Sometimes, a certain level of distrust was shown when services did not show transparently what they offered.

*Because I think this is what happens to all parents, they just don’t have enough time and if they had more time, maybe they would care more about things like this, but especially if they have to search things for a long time, then this is why it fails*.

[Interview 7]

Our experts described their situation as being in high demand and even having to work with waiting lists for their services. According to them, the reason is a shortage of personnel and not saturation of demand. They even raised concerns about their outreach to families in need. Most experts see ignorance of their services as the most important reason that they are not used. They stated that barriers to asking for help are naturally high and that the process of finding help is time-consuming; therefore, many families cannot overcome such a barrier. Additionally, they suggested that families in need are often in denial about their own condition or even consider social and health services as a threat that needs to be controlled. Psychological conditions such as lethargy due to depression constitute another barrier to looking for and taking advantage of services. All experts recognized that a digital tool to navigate families would be helpful in lowering access barriers.

*In our experience, I would say, very paradoxically, the smaller the problem, the higher the parents’ willingness to ask for help and vice versa*.

[Interview 14]

*Well, I think the process of looking for help is complicated for postpartum depression, for example, because until you find someone to take you in, there is just not enough capacity in small cities. I think it would help to have some kind of shortcut to a contact person*.

[Interview 10]

### High Preuse Acceptance of App-Based Intervention

#### Overview

*You see, hardly anybody reaches for a book or magazine or something similar while waiting for something. Usually, you have your phone with you*.

[Interview 6]

This quote shows the importance of mobile devices in our patients’ everyday lives. During our interviews, we saw great acceptance for an app-based intervention on the part of both prospective users and the service providers. As suitable devices, smartphones and handhelds were most often named. Service providers found a computer format more convenient for their daily work.

*As a provider, the computer would be the most appropriate, but I think for most of the users the mobile phone is most appropriate*.

[Interview 16]

We created a mind-map showing the most relevant points concerning the acceptance of our navigator, both from a user and provider standpoint (Multimedia Appendix 2).

#### High Demand for App-Based Navigator

All our prospective users saw the benefit of an app-based navigator to lower the threshold. They especially mentioned how they missed a sense of direction when looking for specific services. Especially, primipara considered their lack of experience as a source of uncertainty throughout their pregnancy. When those people in their surroundings have had little or have had bad experiences, they see the guidance through an app as a source of trust.

*Because especially when you are expecting your first child you know zero of what to expect. There is this big unknown territory*.

[Interview 6]

Not only do prospective users see the wide range of offers and the inherent lack of transparency here as hindering, but experts, too, emphasized the importance of having clear structures and orientation within health care services to ameliorate pre- and postnatal care.

*And then we decide within the situation which help systems are needed. And I think, this is like a jungle for women but also for us midwives*.

[Interview 13]

Another main improvement this study’s participants found in using app-based navigators is time-efficient self-education, which gives them a feeling of security and strengthens their partnerships when sharing new information within the app. Most of our prospective users emphasized that it is important to have one app that combines all aspects of pregnancy, the postpartum period, and early childhood, instead of having a multitude of books and apps for only certain subjects.

*For example, we bought the book Oh I am growing where all development steps are explained because I did not want to have an app, but now the book is actually just sitting on the bookshelf*.

[Interview 7]

Service providers also had a very positive perception of the improvements a future digital navigator could bring. They considered an app where all health-related information could be collected as an improvement in efficiency, making it easier to treat their patients or adequately help their clients. Some of them could envision the app as part of an early warning system, making interventions available sooner to those who need them. Concerning the target group, they thought an app might have a better reach due to the low threshold for gaining information on services and due to the privacy aspect of self-assessing one’s need through psychological check-ups and symptom tracking tools. They stated that a digital navigator might be able to lift the burden from some overcrowded postnatal wards and go hand in hand with hospitals’ psychosocial support concepts. Like our prospective users, they believe that the app can help give a sense of security and orientation throughout this challenging time.

*And I would recommend it because it will convey knowledge to parents, mothers, so as to give a little bit of assurance and orientation in this new phase and an important way to network. In the broadest sense of I know where to turn to when I need it and this will indirectly positively influence the children because mothers will feel more confident in their role as mothers or fathers in their role as fathers*.

[Interview 14]

#### Promoting Factors for an App-Based Navigator

Design-wise, our interviewees found a clear-cut structure with a user-friendly interface, as well as easy-to-understand instructions and messages, to be most agreeable. Both experts and prospective users emphasized that anything that would lower the threshold will enhance user engagement.

*(…)the more I feel at ease, because through colors, font, structure or something else it might leave me with a certain feeling, the more I would be willing to spend time on the app*.

[Interview 6]

Both experts and prospective users expressed the need to have a filter function on the displayed services. Filters should include distance and availability of services to promote an efficient search. Prospective users would also look for ratings or comparison tools for services. They would like to have a direct link to the services and information on whether certain offers fall within their insurance benefits. To contact the services, most users would like to have a direct link to the home page or their email address, or telephone number, and some would even prefer to have a direct chat option.

Prospective users listed the following as important content for the self-education part of the navigator: financial aspects such as how to apply for parental benefits; information on the development of the fetus with individual updates according to the gestational age; information on prenatal care, advice on symptoms during the pregnancy, symptom- and weight-tracking tools; calendar function for all doctors’ and health care services’ appointments and deadlines; checklists for daily questions; information on delivery options and health care providers; support for the postpartum period; and list of postnatal courses, childcare services, information on breastfeeding, activities for babies, and nutritional advice pre- and postpartum.

In addition to the aforementioned content, experts included: relief opportunities for mothers such as family midwives, motherhood nurses, or simple food delivery options; help on finding a midwife and information about their importance; emergency telephone number when dealing with negative feelings after a traumatizing birth or due to postnatal depression; lists of medical doctors and psychologists; lists and information on where to give birth; organizational check lists pre- and postpartum; nutritional and sport advice during pregnancy and nursing period; advice on sleep rhythm and signaling of babies, as well as development milestones; and networking possibilities such as self-help groups.

When asked whether the women wanted to have their partner included in the app, most found it to be helpful. Experts also distinguished the importance of including partners.

*I think this might be even more important than for myself. As a mother, one is a lot closer to the baby, and therefore, it is easier to find out what is wrong with it. Especially when the father is home alone then he has something like a guidebook. It could be a good back-up to look up things*.

[Interview 1]

The last promoting factor that both sides brought up in nearly every interview is the trustworthiness of the information and the contacts that are displayed, and how this might be the key factor for women to continue using and working with the app.

*The maximum of neutrality and the maximum of transparency and the maximum of the maximum of credibility. This is the basic prerequisite, and this needs to be conveyed*.

[Interview 20]

#### Barriers to App Use

Possible reasons for not using such an app were dominated by data protection concerns, and in some form, a fear of becoming dependent on the smartphone. This was connected to time-consuming installation processes or questionnaires, which could lead to user dissatisfaction or even to withdrawal from the intervention. It was also mentioned that if the information was not kept up-to-date, users would stop using the app soon after realizing this.

*I think first and foremost, data protection reasons. I think there are many who might be a little timid in this case. As to maybe their data might be given to third parties or maybe they must disclose too much*.

[Interview 8]

Some participants stated that people might not be well informed or generally did not acknowledge their own need for help. Here, experts found lower education status, language barriers, and psychological conditions to be the cause.

*They think they already know everything and will manage solely on their gut instinct. Otherwise, I do not see a reason because applications are omnipresent and only rarely do people shy away from using them*.

[Interview 10]

Others thought that users might miss the personal contact when dealing with their problems through an app. Experts had doubts in particular about video-based therapy options, which they had already had to incorporate in their daily work due to COVID-19 restrictions.

*Maybe that is just because of anonymity, the nonverbal, the missing conversation*.

[Interview 13]

In general, some assessed that the market for app-based interventions might already be saturated. Prospective users said they would not use an app if it were not free of charge. At the same time, none of the prospective users wanted to have advertisements within the app.

### Digital Health Survey

**Figure 2.**
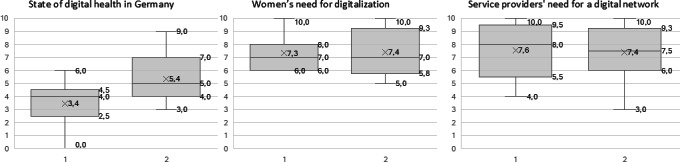
Boxplots of results from the digital health survey; possible answers ranged from 1 to 10 (1 meaning the least and 10 meaning the highest); 1=perspective users, 2=experts; marked within the plot are mean (X), median, and IQRs.

At the end of the interview, we asked our participants some general questions about their opinions concerning digital health in Germany (Multimedia Appendix 1). They indicated that the state of digital health in Germany is at least mediocre. However, experts still had a higher opinion than our prospective users, where one even gave a zero, which was outside the given range of 1‐10. When asked about the need for more digital interlinkage between women and services, both sides estimated that the need is fairly high. Here, opinions did not differ between experts and users (Figure 2). We asked whether not only interlinking providers and users but also providers with providers might be beneficial. All the respondents thought that creating a digital network for service providers would improve access for women. A total of 13 out of 19 respondents thought it would also ameliorate the quality of services, while 6 respondents thought it would not have any effect on the quality. When we asked our service providers specifically, 82% indicated it would enhance their efficiency (Multimedia Appendix 3).

## Discussion

### Improving Access to Psychosocial Services in Germany

This study showed that most women already access information about perinatal services online and have a high willingness to take advantage of general perinatal services, in particular. Barriers consist mainly of a lack of knowledge, time and affordability constraints, and a certain degree of distrust in the qualification and effectiveness of the services. On the other hand, most experts stated that the services they offered are in high demand, and both pregnant women and young mothers reflected this in their interviews. This outcome can be argued in light of the sociodemographic background of our study participants and the region of Heidelberg, where the annual household average income is approximately €24,400 (approximately US $27,469), which is among the highest in Germany [25]. Our multicenter approach to the RCT, involving centers from low-income areas such as Berlin Tempelhof and more rural areas such as Jena, will provide a more representative study population. The next question we asked is whether the demand is high relative to what is being offered and the capacity of services, or whether services are saturated for other reasons, such as underfunding and understaffing. This fact was explicitly mentioned by some of our experts. While stating that their resources are limited, they also saw an urgent need to reach more families in need, suggesting that their services needed improved access points. This finding compares well with similar studies in Germany showing that there is, in fact, a need to improve access to perinatal services for hard-to-reach subgroups [14,26].

There is a significant amount of evidence pointing in the direction of mHealth for improving access to psychosocial treatments [27]. Furthermore, multiple studies showed that mHealth interventions had positive effects on a large range of psychosocial measurements, including self-management of health, acceptance of pregnancy, anxiety, depressive symptoms, perceived stress, mental well-being, coping, and self-efficiency. It also showed that mHealth interventions positively influenced social support from partners or health care providers. The greatest effect was observed in highly vulnerable pregnant women, leading to the conclusion that a perinatal navigator can effectively help vulnerable groups that we are missing right now [28-31]. In prior studies, interlinking women with service providers or enhancing self-help through mHealth interventions seemed to be the key concept. We took this a step further by asking whether it might be beneficial not only to interlink providers and users but also to connect providers with providers. All the respondents thought that access could be facilitated for women by creating a digital network for service providers. Most of the respondents thought it would also improve the quality of services. When we asked our service providers specifically, 82% indicated it would enhance their efficacy. Creating interdisciplinary networks within perinatal care is an ongoing aim for health care providers and has been shown to be beneficial, for example, in mental health settings [32,33]. So far, interprofessional networks have been based on personal meetings and consultations, and to our knowledge, a telemedical network has not yet been implemented within perinatal care. We submit that regional interdisciplinary networks can be created and implemented via a mobile navigator and will improve access to and uptake of perinatal services.

### High Preuse Acceptance Promising for RCT Outcomes

All of our study participants gave positive feedback and showed high preuse acceptance. Most pregnant and postpartum women have already used apps during their pregnancy. Promoting factors for app use from the user’s perspective were time-efficient self-education concerning evidence-based information, positive feedback mechanisms through symptom tracking, direct and easy contact opportunities to regional perinatal and psychosocial services, and networking possibilities. Providers estimated a destigmatization of risk factors, the low threshold for information, and for access to specific services, as well as the chance to better monitor high-risk pregnancy, as success factors for a perinatal app-based navigator. On the subject of perinatal and postpartum depression, a study from Varma et al [34] showed comparable results on acceptance and utility. Especially during the COVID-19 pandemic, we saw an increased interest in the subject of telemedical monitoring. Several studies showed that virtual visits and communication through telemedicine are highly accepted on both the patient and provider sides of health care [35-37]. Barriers to app use expressed by future users were mainly unawareness, data protection concerns, complexity of app use, hidden costs, or in-app advertisements, as well as the already high number of apps on the market. Providers added language barriers, low education profile of users, and mismatch of app design, denial of necessity, and psychological lethargy, as well as a general digital skepticism, to this list. Similar barriers to telehealth uptake, including level of education, telehealth literacy, and unawareness, were found in a multinational review by Kruse et al [38]. What differs from other studies is our family-centered approach in a multidisciplinary context, allowing multiple entry points, as well as a personalized solution for women and young families navigating through this phase.

### Limitations

There are several limitations to this study. The first and perhaps greatest limitation concerns our study participants’ socioeconomic status and level of education, which is only partly representative of the foreseen cohort of women with psychosocial risk factors from hard-to-reach socioeconomic niches. Furthermore, all participants were from the same geographic area, which might lead to results not being generalizable nationally. Nonetheless, tailoring the app to the needs of prospective users and health care providers should help the intervention to be more successful. Testing the mHealth intervention in a multicenter setting will help with representative data. Another limitation is the small sample size, which is characteristic of qualitative research methodology. To validate the data, we used researcher triangulation. Interviews were conducted by two investigators, and data interpretation was done in a team of three. By introducing a small survey with closed-ended questions, we were able to triangulate common notions on digital health.

### Conclusions

This study demonstrated high acceptance of an app-based perinatal navigator both from the users’ perspective and the psychosocial and health care providers’ points of view. It also showed that creating better networks between users and providers, but also between providers and providers, will help improve access and quality of care. Feedback on requirements and content, as well as possible barriers, informed our concept and helped in creating all parts of the self-education guide. All information was curated by a team of health care professionals from different specialties, including midwives, gynecologists, psychologists, and pediatricians. The results from this study underscore the necessity for a multicenter RCT on the perinatal navigator.

## Supplementary material

10.2196/66658 Multimedia Appendix 1 Interview guide.

10.2196/66658 Multimedia Appendix 2 Mind map of expert and user perspectives.

10.2196/66658 Multimedia Appendix 3 Digital health survey results.

## Abbreviations

mHealth: mobile health
RCT: randomized controlled trial
