# Supplementary material for: Preuse Acceptance of a Family-Centered, Need-Based, and Interprofessional Perinatal Care Mobile Health Intervention: Exploratory Study
Source: JMIR Hum Factors. 2025 Jun 12;12:e66658. doi: 10.2196/66658 (PMC12202979; doi:10.2196/66658)
Supplement: Multimedia Appendix 1 [file humanfactors-v12-e66658-s001.docx]

**Interview guide:**

1. The interview will be recorded electronically and then transcribed in pseudonymized form. Upon completion, the audio recording will be deleted. Information about places or people you provide in the interview will be anonymized. When it comes to interviews, there are no right or wrong opinions. We are interested in your personal assessments and experiences. Do you agree?
2. Then let's start with the questions about you. Can you tell us your age?
3. Your highest professional qualification or educational qualification.
4. Then the status of your partnership.
5. Do you already have children?
6. What week of pregnancy are you in right now? How old is your child?
7. Do you have any pre-existing conditions?
8. Have there been any complications so far?
9. What channels do you currently use to get in touch with service providers for pregnant women or young families?
10. Are you currently using offers for pregnant women or young families? And if so, which ones?
11. Can you name a few reasons that would perhaps speak for you against taking advantage of certain offers?
12. Could digital services, such as an online portal or an app, simplify access to offers? Maybe also lower the threshold?
13. What do you think an app would have to do to improve the medical or psychosocial care of pregnant, stressed mothers and families and also to be accepted?
14. What incentives do you think should be offered so that pregnant mothers are willing to fill out a questionnaire or seek help online?
15. Then imagine that there is the possibility of making pregnant women and young families aware of counseling services at an early stage with the help of a digital application, both regionally and nationally. What offers should such a digital navigator contain?
16. What function or communication structure of an app would you like to see in order to get in touch with the service providers? So, for example, is there only the phone number or should there be a chat function stored or the website linked?
17. What should then be considered so that the offers also reach pregnant women and young families and can then also be taken advantage of?
18. In which area would you have the greatest need for support? What topics would you be most interested in?
19. Would you recommend such an online portal that provides offers around the period of pregnancy and childbirth to a pregnant friend or colleague? If yes or no? Why each?
20. What difficulties or reasons do you think are conceivable that would prevent pregnant mothers or families from using such an app?
21. What requirements should be met with regard to the graphical presentation of the content offered or the handling of the app?
22. Would you like your partner to be included in the app? If so, how would you imagine it?
23. Which device would be most suitable for you as a user?
24. In general, what do you think about the possibility of using smartphone applications in medical treatment, in checking health status. and also to improve quality of life in the future?
25. Do you have any recommendations for us right now, any thoughts you'd like to share before moving on to the next section?

We still have a small digitization questionnaire with closed questions. Answers to the next questions range from 1 to 10, where 1 is very bad and 10 is very good.

1. In your opinion, how good is the current state of digitalization in the healthcare sector?
2. In your opinion, how great is the need for digital networking of healthcare providers (doctors, midwives, therapists...) and pregnant women/mothers/families on the part of pregnant women?
3. In your opinion, how great is the need for digital networking of healthcare providers and pregnant women and mothers on the part of healthcare providers?
4. In your opinion, how great is the need for digital networking among healthcare providers?
5. In your opinion, what influence would digital networking of healthcare providers have on access to medical care?
6. In your opinion, what influence would digital networking of healthcare providers have on the quality of care for pregnant women/mothers/families?
7. In your opinion, what influence would a digital networking of service providers in the healthcare sector have on the efficiency of their work area?
8. In your opinion, what influence would the introduction of the digital maternity record have on the care of pregnant women/mothers?
9. In which area do you see the greatest need for information among (expectant) mothers and young families?Examples:

o Child development

o Nutrition

o Sport

o Mental health

o Symptom monitoring

o Birth planning

o Or something else entirely

1. Do you have anything else to add? Otherwise we would be finished with the questions.
