# Supplementary figures and images for: Preuse Acceptance of a Family-Centered, Need-Based, and Interprofessional Perinatal Care Mobile Health Intervention: Exploratory Study
Source: JMIR Hum Factors. 2025 Jun 12;12:e66658. doi: 10.2196/66658 (PMC12202979; doi:10.2196/66658)

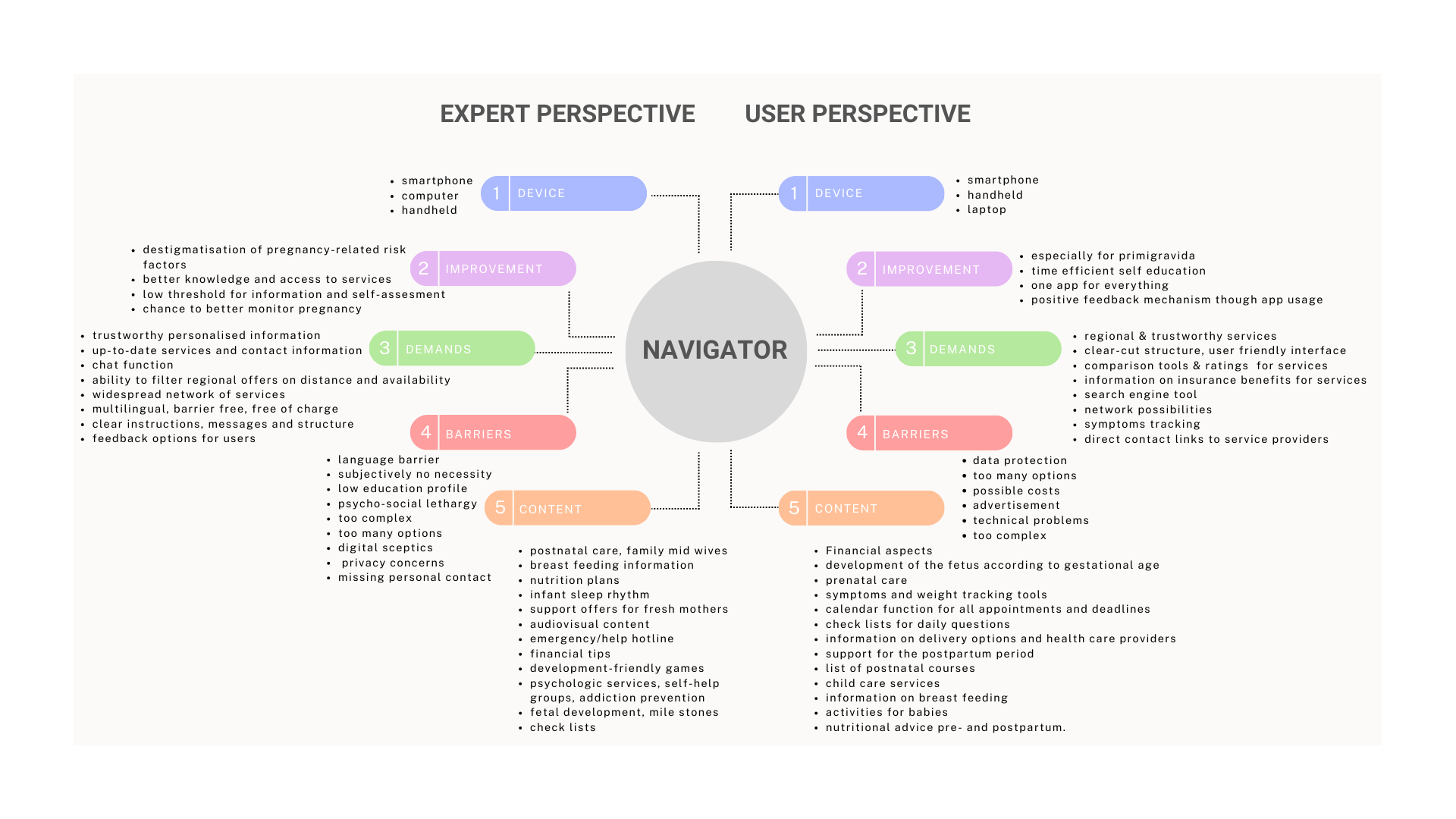

Supplement: Multimedia Appendix 2 [file humanfactors-v12-e66658-s002.png]
