# Supplementary material for: Preuse Acceptance of a Family-Centered, Need-Based, and Interprofessional Perinatal Care Mobile Health Intervention: Exploratory Study
Source: JMIR Hum Factors. 2025 Jun 12;12:e66658. doi: 10.2196/66658 (PMC12202979; doi:10.2196/66658)
Supplement: Multimedia Appendix 3 [file humanfactors-v12-e66658-s003.docx]

| InterviewID | Group | Digitalization | Demand pregnancy | Demand services | Service to service | Access | Quality | Efficiency | Digital motherspass |
| --- | --- | --- | --- | --- | --- | --- | --- | --- | --- |
| 1 | 1 | 3 | 8 | 10 |  | 1 | 2 |  | 1 |
| 2 | 1 | 4 | 8 | 6 |  |  |  |  |  |
| 3 | 1 | 4 | 7 | 8 |  | 1 | 2 |  | 2 |
| 4 | 1 | 4 | 8 | 8 |  | 1 | 1 |  | 1 |
| 5 | 1 | 5 | 6 | 5 |  | 1 | 1 |  | 1 |
| 6 | 1 | 0 | 6 | 9 |  | 1 | 1 |  | 2 |
| 7 | 1 | 2 | 10 | 10 |  | 1 | 1 |  | 1 |
| 8 | 1 | 3 | 7 | 8 |  | 1 | 1 |  | 1 |
| 9 | 1 | 6 | 6 | 4 |  | 1 | 2 |  | 1 |
| 10 | 2 | 9 | 5 | 3 | 10 | 1 | 1 | 1 | 3 |
| 11 | 2 | 7 |  |  | 8 | 1 | 1 | 2 | 1 |
| 12 | 2 | 4 | 10 | 10 | 7 | 1 | 1 | 1 | 1 |
| 13 | 2 | 5 | 7 | 10 | 10 | 1 | 1 | 1 | 1 |
| 14 | 2 | 5 | 10 | 7 | 8 | 1 | 2 | 1 | 1 |
| 15 | 2 | 3 | 6 | 6 | 10 | 1 | 1 | 3 | 3 |
| 16 | 2 | 4 | 7 | 7 | 9 | 1 | 2 | 1 | 1 |
| 17 | 2 | 5 | 9 | 9 | 9 | 1 | 1 | 1 | 1 |
| 18 | 2 | 4 | 6 | 6 |  | 1 | 1 | 1 | 1 |
| 19 | 2 | 4 | 9 | 8 | 8 | 1 | 2 | 1 | 1 |
| 20 | 2 | 9 | 5 | 8 | 9 | 1 | 1 | 1 | 2 |
